# A one-year follow-up study of treatment-compliant suicide attempt survivors: relationship of *CYP2D6-CYP2C19* and polypharmacy with suicide reattempts

**DOI:** 10.1038/s41398-022-02140-4

**Published:** 2022-10-18

**Authors:** Eva M. Peñas-Lledó, Sebastien Guillaume, Fernando de Andrés, Ana Cortés-Martínez, Jonathan Dubois, Jean Pierre Kahn, Marion Leboyer, Emilie Olié, Adrián LLerena, Philippe Courtet

**Affiliations:** 1 INUBE Biosanitary University Research Institute, University of Extremadura, Badajoz, Spain; 2 University of Extremadura Medical School, Badajoz, Spain; 3 IGF, CNRS, INSERM, Univ. Montpellier, Montpellier, France; 4 Department of Emergency Psychiatry and Acute Care, Lapeyronie Hospital, CHU Montpellier, 34090 Montpellier, France; 5 Université de Lorraine, Nancy, France, Clinique Soins-Etudes de Vitry le François, Fondation Sant´e des Etudiants de France (FSEF), Paris, France; 6 FondaMental Foundation, Créteil, France; 7 Univ Paris Est Créteil, INSERM U955, IMRB, Translational NeuroPsychiatry Laboratory, Créteil, France; 8 AP-HP, Hôpitaux Universitaires Henri Mondor, Département Médico-Universitaire de Psychiatrie et d’Addictologie (DMU IMPACT), Fédération Hospitalo-Universitaire de Médecine de Précision en Psychiatrie (FHU ADAPT), Créteil, France; 9 CIBERSAM, Instituto de Salud Carlos III, Madrid, Spain

**Keywords:** Predictive markers, Scientific community

## Abstract

This study of a cohort of 1-year treatment-compliant survivors of a suicide attempt examined for the first time whether a high *CYP2D6-CYP2C19* metabolic capacity (pharmacogenes related to psychopathology, suicide, and attempt severity) and/or polypharmacy treatments predicted repeat suicide attempts, adjusting for sociodemographic and clinical factors as confounders. Of the 461 (63% women) consecutively hospitalized patients who attempted suicide and were evaluated and treated after an index attempt, 191 (67.5% women) attended their 6- and 12-month follow-up sessions. Clinicians were blinded to the activity scores (AS) of their genotypes, which were calculated as the sum of the values assigned to each allele (CYP2C19 *2, *17; CYP2D6 *3, *4, *4xN, *5, *6, *10, *wtxN*). No differences were found in polypharmacy prescription patterns and the variability of *CYP2D6* and *CYP2C19* genotypes between adherents and dropouts, but the formers were older, with a higher frequency of anxiety and bipolar disorders and fewer alcohol and substance use disorders. The risk of reattempts was higher for *CYP2D6* ultrarapid (AS > 2) metabolizers (β = 0.561, *p* = 0.005) and violent suicide survivors (β = −0.219, *p* = 0.042) if the attempt occurred during the first 6-month period, individuals with an increased number of MINI DSM-IV Axis I mental disorders (β = 0.092, *p* = 0.032) during the second 6-month period and individuals with a combined high *CYP2D6-CYP2C19* metabolic capacity (AS > 4) (β = 0.345, *p* = 0.024) and an increased use of drugs other than antidepressants, anxiolytics-depressants and antipsychotics-lithium (β = 0.088, *p* = 0.005) in multiple repeaters during both periods. CYP2D6 and CYP2C19 rapid metabolism and polypharmacy treatment for somatic comorbidities must be considered to prevent the severe side effects of short-term multiple suicide reattempts after a previous attempt.

## Introduction

A history of suicide attempts appears to be the strongest predictor of future reattempts [1, 2]. However, identifying individuals at high risk for suicide is clinically difficult [3]. This identification is particularly important for suicide prevention purposes and within the first year following a suicide attempt [4] to assist and stratify treatment selection. Approximately 15% of patients will reattempt suicide during the year following an index attempt [4]. In addition, prospective studies show that ~10% of the survivors of a suicide attempt ultimately die by suicide [5]. However, the risk of a subsequent suicidal death in reattempters is substantially reduced if follow-up appointments are scheduled [6]. Although repeaters of suicide attempts appear to be heavy consumers of health resources and pose a challenge to clinicians [7, 8], little is known about the risk factors for those who continue attempting even while keeping these appointments.

Most research on risk factors for multiple suicide attempts focuses on sociodemographic and clinical factors [9], but little research has been devoted to pharmacotherapeutic-related risk factors, such as polypharmacy prescription patterns and pharmacogenetic factors related to interindividual variability in drug failure.

Pharmacological options for treating short-term suicidal risk have only recently become available [10]. Indeed, the role of antidepressants in suicide prevention is debated, and high-risk patients with depression have been argued to respond less well to short-term antidepressants [11, 12]. Consistently, clinicians may use augmentation strategies to optimize the antidepressant response and search for an anti-suicide effect (e.g., lithium and antipsychotics).

A common psychiatric prescription practice in cases of treatment resistance is to concomitantly use multiple drugs (polypharmacy) [13, 14]. However, concerns about the risks of polypharmacy are growing. Evidence shows that the risk of potential adverse outcomes due to drug‒drug interactions, hospital admissions, health care costs and nonadherence to therapy increase as the number of drugs a patient takes increases [15, 16]. Furthermore, polypsychopharmacotherapy in psychiatric patients has been retrospectively related to a history of suicide attempts [17, 18] and suicide risk [19].

CYP2D6 and CYP2C19 are two highly polymorphic P450 enzymes involved in the metabolism of many central nervous system medications used to prevent suicide, such as most antidepressants [20], antipsychotics [21] or anxiolytics [22], and this polymorphism may lead to variability in drug response during treatment. Consistently, a poorer response to antidepressants has been observed in individuals carrying multiplications of *CYP2D6* active genes [23–27] or *CYP2C19* enhanced-activity associated alleles [28].

Variability in CYP2D6 activity has also been involved in the metabolism of endogenous substrates, which may explain differences with regard to suicidal behavior. The number of *CYP2D6* active genes has been related to suicide death [29], a lifetime history of suicide attempts [30], suicide risk [31], and the severity of the objective circumstances mostly related to the preparation of the suicide attempt [32]. Similarly, *CYP2C19* has been shown to be expressed in the human foetal brain and to explain differences in the brain development and affective behavior of transgenic mice carrying the human *CYP2C19* gene [33]. Consistently, high *CYP2C19* enzymatic capacity has been associated with higher suicidality in individuals with depression who attempt suicide [34], whereas its absence was associated with lower levels in state markers of suicide, such as lower depressive symptoms [35], a lower prevalence of major depressive disorder and depression severity and increased bilateral hippocampal volume in healthy cohorts [34]. Likewise, elevated *CYP2C19* expression was related to depression, reduced hippocampal volume and the impairment of hippocampal serotonin homeostasis [34].

The aim of the present study was to prospectively evaluate for the first time whether a separate and/or combined high metabolic capacity of *CYP2D6* and *CYP2C19* and the number of prescribed drugs predict repeat suicide attempts within a year of the last index in treatment compliance survivors (who keep the scheduled appointments). The ultimate purpose is to develop stratified preventive pharmacotherapeutic programs.

## Materials and methods

### Participants

**Fig. 1:**
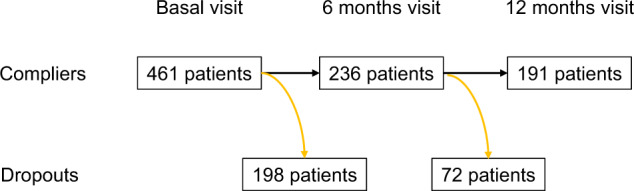
Flowchart of the participants of the study. The number of patients included at each phase of the evaluation period (Compliers), as well as of patients who did not attend (Dropouts) is detailed.

A total of 461 patients consecutively hospitalized and survivors of a current suicide attempt were evaluated in a specialized unit of the Montpellier University Hospital (*N* = 203), Nancy (*N* = 115), and Créteil (*N* = 143). Of the 191 patients, 191 attended their 6- and 12-month follow-up sessions (Fig. 1).

All participants gave written informed consent, as approved by the local ethics committee (CPP Sud Mediterran´ee IV, CHU Montpellier, France) after receiving information on the study.

### Sociodemographic and clinical evaluation

Patients were evaluated for sex, age, civil status, years of schooling, current DSM-IV Axis I mental disorders as assessed by the MINI-International Neuropsychiatric Interview and current tobacco smoking.

### Evaluation of suicide

Patients were longitudinally evaluated for suicide attempts at two times (6 and 12 months) within the first year of the index suicide attempt. All patients were clinically evaluated on the different variables in relation to the repetition of suicide attempts during the first and second 6-month periods. A suicide attempt was defined as ‘a nonfatal self-directed potentially injurious behavior with any intent to die as a result of the behavior. A suicide attempt may or may not result in injury’ [36].

In addition, patients were also asked whether this was their first suicide attempt or if they had a personal history of suicidal attempts at baseline. The index suicide attempt was also classified as violent if it involved firearms, drowning, deep-cutting, jumping or hanging, according to Asberg’s criteria [37]. In addition, the Life History of Aggression (LHA) [38], the Scale for Suicide Ideation (SSI) [39], and the Suicide Intent Scale (SIS) [40, 41] were also evaluated at baseline.

### *CYP2D6* and *CYP2C19* genotyping and the combined metabolic score

As previously described [42], patients were genotyped for CYP2C19*2 and CYP2C19*17 as well as for CYP2D6*3, *4, *4xN, *5, *6, CYP2D6*10 and *wtxN* [32, 42] in the Clinical Research Center of Extremadura University Hospital and Medical School.

Additionally, a metabolic score for *CYP2D6* and *CYP2C19* genotypes and for their combination, which was based on the sum of the *CYP2D6* and *CYP2C19* activity scores (AS) [43–46], was calculated for each subject, as previously described [47]. Because they are related to null enzyme activity, the following allelic variants were given an activity score of zero: CYP2D6 *3, *4, *4xN, *5, *6 and CYP2C19*2. The wild-type (*wt*) allelic variants for both CYPs received a score of one because they are related to normal enzyme activity, whereas *CYP2D6 wtx2* and CYP2C19*17 received a score of 2 for their relationship to rapid enzyme activity. Therefore, the proposed “combined metabolic score” or AS could possibly range from 0 to 8 in one-point increments, with higher scores indicating higher metabolic capacity. Finally, as previously described [47], once the sum of the combined CYP2D6-CYPC19 activity scores was calculated, individuals were classified into three groups characterized by having a combined “high” drug metabolic capacity score above 4 (AS > 4), “medium” (AS = 4) or “low” (AS < 4).

This approach serves to illustrate the assumed functional effects of the combined genotypes for these two polymorphic genes with regard to the number of active alleles, which are assumed to influence drug metabolic activity (Table 1).

**Table 1:** Frequency of *CYP2D6* and *CYP2C19* genotypes.

Gene	Baseline visit; *N* = 461 (95% CI)	Dropouts; *N* = 270 (95% CI)	Adherent; *N* = 191 (95% CI)
***CYP2D6***			
PMs			
*3/*4	0.009 (0.003–0.022)	0.007 (0.002–0.027)	0.010 (0.003–0.037)
*4/*4	0.039 (0.025–0.061)	0.037 (0.020–0.067)	0.042 (0.021–0.080)
*4/*4xN	0.002 (0.000–0.012)	0.004 (0.001–0.021)	NF
*4/*5	0.022 (0.012–0.039)	0.022 (0.010–0.048)	0.021 (0.008–0.053)
*4/*10	0.002 (0.000–0.012)	0.004 (0.001–0.021)	NF
*5/*5	0.004 (0.001–0.016)	0.004 (0.001–0.021)	0.005 (0.001–0.029)
*5/*6	0.002 (0.000–0.012)	NF	0.005 (0.001–0.029)
NMs			
*wt/wt*	0.518 (0.473–0.564)	0.507 (0.448–0.566)	0.534 (0.463–0.603)
wt/*3	0.017 (0.009–0.034)	0.015 (0.006–0.038)	0.021 (0.008–0.053)
wt/*4	0.215 (0.180–0.254)	0.219 (0.173–0.272)	0.209 (0.158–0.273)
wt/*4xN	0.004 (0.001–0.016)	0.007 (0.002–0.027)	NF
wt/*5	0.050 (0.033–0.074)	0.059 (0.037–0.094)	0.037 (0.018–0.074)
wt/*6	0.019 (0.010–0.037)	0.026 (0.013–0.053)	0.010 (0.003–0.037)
wt/*10	0.024 (0.013–0.042)	0.030 (0.015–0.057)	0.016 (0.005–0.045)
wtx2/*3	0.002 (0.000–0.012)	0.004 (0.001–0.021)	NF
wtx2/*4	0.015 (0.007–0.031)	0.004 (0.001–0.021)	0.031 (0.014–0.067)
wtx2/*10	0.002 (0.000–0.012)	0.004 (0.001–0.021)	NF
UMs			
*wt/wtxN*	0.052 (0.035–0.076)	0.048 (0.028–0.081)	0.058 (0.032–0.100)
***CYP2C19***			
PMs			
*2/*2	0.0152 (0.007–0.031)	0.019 (0.008–0.043)	0.010 (0.003–0.037)
NMs			
wt/*2	0.200 (0.166–0.238)	0.189 (0.147–0.240)	0.215 (0.162–0.278)
*wt/wt*	0.427 (0.383–0.473)	0.437 (0.379–0.497)	0.414 (0.346–0.484)
*2/*17	0.089 (0.066–0.118)	0.089 (0.060–0.129)	0.089 (0.056–0.138)
UMs			
wt/*17	0.252 (0.214–0.293)	0.256 (0.207–0.311)	0.246 (0.190–0.312)
*17/*17	0.017 (0.009–0.034)	0.011 (0.004–0.032)	0.026 (0.011–0.060)

*PMs* poor metabolizers (AS = 0), *NM* normal metabolizers (1 ≤ AS ≤ 2), *UMs* ultrarrapid metabolizers (AS > 2), *NF* not found.

### Evaluation of pharmacological treatment at baseline, 6 and 12 months

The total number of drugs at the baseline and follow-up visits (6 and 12 months) was calculated and stratified into the mean number of antidepressants, anxiolytics and depressants (sedatives and hypnotics), antipsychotics and lithium, and other drugs (Table 2).

**Table 2:** Evaluation of pharmacological treatment at baseline (compliers and dropouts), for dropouts and compliers at 6 and 12 months.

	Baseline compliers (*N* = 191)	Baseline dropouts (*N* = 270)	Compliers 6 months (*N* = 191)	Compliers 12 months (*N* = 191)
Number of drugs	4.19 ± 2.17	4.13 ± 2.22	2.92 ± 1.95	2.79 ± 2.07
Antidepressants	0.70 ± 0.49	0.72 ± 0.51	0.69 ± 0.55	0.74 ± 0.57
Antipsychotics, lithium	0.73 ± 0.80	0.62 ± 0.76	0.44 ± 0.63	0.37 ± 0.58
Anxiolytics, depressants	0.88 ± 0.70	0.94 ± 0.74	0.74 ± 0.83	0.65 ± 0.85
Other drugs	1.88 ± 1.91	1.85 ± 1.84	1.05 ± 1.35	1.02 ± 1.43

Data are expressed as mean ± SD.

### Statistical analysis

First, a univariate analysis based on t (Student’s) hypothesis testing or a generalized linear mixed model (GLMM) and a Pearson’s *χ*^2^ test or Fisher’s exact test were carried out to identify the respective independent quantitative and qualitative variables and covariables significantly related to a repeated suicide attempt within the first or/and second 6-month period after the index event in comparison to individuals who did not have such an attempt. As mentioned above, the variables were sex, age, civil status, years of schooling, current MINI DSM-IV Axis I mental disorders and their total number, current smoking status, LHA, SSI, SIS, violent index suicide attempt, previous number of suicide attempts, pharmacogenetic risk factors and number of drugs for treatment.

Three linear models were constructed to assess the individual and collective impact of the significant variables of the initial univariate analysis on the criterion variable. The following breakdown criterion variables were predicted for three different groups of patients: patients who reattempted suicide during the first or second 6-month period after the index attempt or during both time periods within the year.

Two-tailed *p* values <0.05 were considered statistically significant. Data were analysed using SPSS version 23 (IBM Corp. Released, Armonk, NY).

## Results

### Main demographic and clinical data of the baseline sample

The 461 patients consecutively hospitalized during 2010–2011 and the survivors of a current suicide attempt were 62.9% women with a mean age of 42.6 ± 13.0 years and diverse civil status (*n* = 127, 27.6% single; *n* = 202, 43.8% married; *n* = 34, 7.4% separated; *n* = 85, 18.4% divorced and *n* = 13, 2.8% widowed; 12.2 ± 2.9 years of schooling). Notably, 68.3% (*n* = 315) were smokers, and the majority of the population had more than two (*n* = 183, 39.7%) or two (*n* = 183, 39.7%) DSM-IV Axis I mental disorders; a minority had been diagnosed with one disorder (*n* = 73; 15.8%) or had not been diagnosed (*n* = 22; 4.8%). For almost half of the population (*n* = 206; 44.7%), the suicide attempt was their first attempt, and one fourth (*n* = 112; 24.3%) attempted suicide by violent means. The mean scores were 5.82 ± 5.04, 22.95 ± 8.86, and 16.72 ± 5.68 on the LHA, SSI and SIS scales, respectively.

No differences were found in the variables across the hospital centers of origin.

### Sociodemographic and clinical differences between compliers and dropouts

The 191 patients who attended their 6- and 12-month follow-up sessions presented no differences in gender (*χ*^2^(1) = 2.999 *p* = 0.083), civil status (χ^2^(4) = 8.595, *p* = 0.072) or years of schooling (*t* (389.718) = −1.545, *p* = 0.123) compared to the remaining patients evaluated at baseline that dropped out at any moment (*n* = 270). However, this subpopulation of compliers was older (mean age 45.14 ± 12.44 years) than the remaining cohort (40.81 ± 13.11 years) (*t* (459) = −3.357, *p* < 0.05) and included a lower frequency of smokers (61.8% compliers vs. 73.0% dropouts: *χ*^2^(1) = 6.463, *p* = 0.011, *Z*_res_ = 2.5). In addition, the frequencies of alcohol abuse (26.7% vs. 36.1%; *χ*^2^(1) = 4.491, *p* = 0.034, *Z*_res_ = −2.1) and substance use disorders (11.5% vs. 24.3%; *χ*^2^(1) = 11.775, *p* = 0.001, *Z*_res_ = −3.4) were lower, but the frequencies of anxiety (98.6% vs. 86.9%; *χ*^2^(1) = 15.556, *p* < 0.001, *Z*_res_ = 3.9) and bipolar disorders (29.5% vs. 18.6%; *χ*^2^(1) = 7.305, *p* = 0.007, *Z*_res_ = 2.7) were higher, although no differences were found in the number of DSM-IV Axis I disorders.

Compliers also presented an older mean age at the onset of suicide attempt (37.57 ± 14.88 years vs. 34.08 ± 14.74 years; *t* (459) = −2.490, *p* = 0.013). No significant differences were found between the compliers and dropouts in the number of suicide attempts (*t* (456) = 0.222, *p* = 0.824), history of violent suicide attempts (*χ*^2^(1) = 0.008, *p* = 0.929), LHA (*t* (453) = 0.712, *p* = 0.477), SIS total (*t* (441) = 1.171, *p* = 0.242) and SSI (*t* (451) = 1.134, *p* = 0.257).

Finally, there were no significant differences in the prescribed number of drugs at baseline (Table 2) or in the *CYP2D6* and *CYP2C19* alleles, genotypes, and phenotypes distribution (Table 1) between compliers and dropouts.

### Number of prescribed drugs

Significant differences were found during the follow-up when comparing the total number of drugs taken at baseline (*F* = 36.985, *p* < 0.05), which was higher than at the 6- and 12-month periods, for which the number of prescribed drugs taken were similar (Table 2).

### *CYP2D6* and *CYP2C19* alleles and phenotypes

The population complied with the expected Hardy-Weinberg equilibrium for all *CYP2D6* and *CYP2C19* alleles, except for CYP2C19*17 in both groups (*p* < 0.05). The population that attended the scheduled treatment sessions (*n* = 191) included 8.4% PMs and 5.8% UMs for *CYP2D6* and 1% PMs and 27.2% UMs for *CYP2C19*.

With regard to the three categories defined according to the combined *CYP2D6-CYP2C19* metabolic scores, 36 (18.8%) showed a “high” capacity (AS > 4), 76 (39.6%) a “medium” capacity (AS = 4), and 80 (41.7%) a “low” capacity (AS < 4).

### Repeated suicide attempt within the year of the index attempt

The number of individuals who reattempted suicide at any time was 38 (19.9%). Of them, 14 (7.3%) reattempted between the index attempt and the first follow-up session, 12 (6.3%) reattempted between the first and second follow-up sessions, and 12 (6.3%) reattempted because they repeated at least twice within the first follow-up year and at least once during each follow-up period.

### Association between the above clinical variables and the repetition of suicide attempts

The variables significantly related to a repeated suicide attempt during the first follow-up period were the number of MINI DSM-IV Axis I mental disorders (*t* (189) = −2.103, *p* = 0.037), index violent suicide method (Fisher, *p* = 0.024), LHA (*t* (188) = −2.322, *p* = 0.021), and *CYP2D6* AS (*χ*^2^(2) = 14.498, *p* = 0.001).

The variables related to repetition of the attempt during the second follow-up period were bipolar disorder (Fisher, *p* = 0.017), depression disorder (Fisher, *p* = 0.032), alcohol abuse disorder (Fisher, *p* = 0.017) and a higher number of current MINI DSM-IV Axis I mental disorders (*t* (189) = −2.775, *p* = 0.006).

Finally, the variables related to multiple suicide reattempts were the total number of prescribed drugs (*t* (189) = −3.934, *p* < 0.001) and other drugs (*t* (189) = −3.955, *p* < 0.001) at baseline and the number of total drugs (*t* (189) = −3.809, *p* < 0.001) and of anxiolytics and depressants prescribed at 6 months (*t* (11.787) = −2.546, *p* = 0.026), AS *CYP2C19* (*χ*^2^(2) = 10.082, *p* = 0.006) and the combined *CYP2D6-CYP2C19* metabolic score (*χ*^2^(2) = 13.183, *p* = 0.001).

Regression analyses showed that the risk of suicide reattempts in the very first six months after the index attempt was more likely in *CYP2D6* UMs (β = 0.561, *p* = 0.005) and if the suicide method was violent (β = −0.219, *p* = 0.042). The risk of reattempts during the second 6-month follow-up period correlated with the number of current MINI DSM-IV Axis I mental disorders at baseline (β = 0.092, *p* = 0.002). Finally, the risk of multiple reattempts was related to the number of other drugs at baseline (β = 0.088, *p* = 0.005) and a high *CYP2D6-CYP2C19* metabolic capacity (β = 0.345, *p* = 0.024).

## Discussion

The present study aimed to identify whether understudied biological and pharmacotherapeutic risk factors, such as pharmacogenetic polymorphisms and the number of prescribed medications[7], predicted repeat suicide attempts at 6 and/or 12 months after the index attempt. This target is relevant, as individuals who continue to attempt suicide during the first year immediately after a suicide attempt represent a major clinical challenge because of their high risk [4, 7]. However, little is known about this population [8]. Although the cumulative attendance rate at follow-up clinical sessions was lower than the rate of patients who dropped out (58%), a similar rate was found in another study [48] with a similar design. Of note, the differences between patients who attended follow-up sessions and patients who dropped out were in sociodemographic and psychopathology-related variables, but the *CYP2D6* and *CYP2C19* genotype and phenotype frequencies or number of drugs did not differ between these groups. Therefore, the results regarding these variables could be generalized.

The main findings showed that the risk of repeat suicide attempts after an index attempt increased at 6 months in patients with an ultrarapid *CYP2D6* metabolic capacity and in patients who made a violent attempt. At 12 months, the risk increased in patients with a greater number of mental disorders. Finally, the risk of repeat suicide attempt increased in both follow-up periods for patients with a high combined metabolic capacity for the two pharmacogenes studied and for patients who used of a greater number of drugs other than antidepressants, anxiolytics or depressants and antipsychotics or lithium at baseline.

The aforementioned increased risk of a suicide attempt during the first 6 months of the index attempt for patients with greater number of active CYP2D6 genes is consistent with previous findings. For example, the proportion of individuals with a high activity or metabolic capacity for CYP2D6 was higher in a Swedish forensic population who died by suicide than in those who died by natural causes [29]. This proportion was also higher in a population of patients with eating disorders and a lifetime history of suicide attempts [30], in individuals at risk for suicide [31] and in individuals with an increased severity of the objective circumstances related to the suicide attempt and its preparation [32]. In addition, patients who exhibited *CYP2D6* UM and received monotherapy treatment with CYP2D6 antidepressant substrates ceased treatment early or have been shown not to benefit from CYP2D6 antidepressant substrates [27]. These findings could apply to patients exhibiting CYP2D6 UM who did not keep their scheduled sessions in the present study.

Other studies of large samples have also found that the use of a violent method [49], psychiatric comorbidities and the number of mental disorders [50, 51] are risk factors for suicide reattempts.

Finally, this study highlighted that the number of concurrent prescriptions, the type of drug prescribed, and a high combined *CYP2D6-CYP2C19* metabolic capacity prospectively increase the risk of multiple reattempts during both the first and second follow-up periods within a year of the initial attempt. Accordingly, polypharmacy is known to increase the risk of adverse outcomes due to drug‒drug interactions [15, 16], and polypharmacy has been retrospectively related to a history [17, 18] and risk of suicide attempts [19]. Polypharmacy includes drugs commonly used to prevent suicide (e.g., antidepressants, anxiolytics and depressants, antipsychotics, and lithium) and additional drugs the patient might be commonly taking. For example, active substances, such as calcium-channel blockers and beta-blockers, antidiabetics, and analgesics, might share metabolic pathways with the drugs used to prevent suicide and/or might be involved in suicide poisoning [52]. Particularly, opioids are prodrugs that need CYP2D6 to convert them into the effective analgesic, morphine, and patients exhibiting CYP2D6 UM are at increased risk of death by suicide and intoxication.

A high *CYP2D6-CYP2C19* metabolic capacity might be related to multiple reattempts due to drug treatment failure in patients taking substrates for these enzymes. Furthermore, ahigh *CYP2D6-CYP2C19* metabolic capacity was previously shown to be related to the severity (or increased fatality likelihood) of the suicide attempt [47].

In short, CYP2D6 and CYP2C19 ultrarapid metabolism and polypharmacy factors must be taken into consideration to prevent short-term multiple suicide reattempts. Future studies warrant further research to identify the drug‒drug interactions and pharmacogenetic profiles that are more likely to predict repeated suicide attempts. Furthermore, the clinical conditions influencing drug kinetics and response (renal clearance, liver function, etc.) must also be considered.

## References

[1] Menon V (2013). Suicide risk assessment and formulation: an update. Asian J Psychiatry.

[2] Turecki G, Brent D (2016). Suicide and suicidal behaviour. Lancet.

[3] Harris EC, Barraclough B (1997). Suicide as an outcome for mental disorders. Br J Psychiatry.

[4] Zahl DL, Hawton K (2004). Repetition of deliberate self-harm and subsequent suicide risk: long-term follow-up study of 11 583 patients. Br J Psychiatry.

[5] Osváth P, Kelemen G, Erdös MB, Vörös V, Fekete S (2003). The main factors of repetition: review of some results of the Pecs center in the WHO/EURO multicentre study on suicidal behaviour. Crisis.

[6] Bostwick JM, Pabbati C, Geske JR, McKean AJ (2016). Suicide attempt as a risk factor for completed suicide: Even more lethal than we knew. Am J Psychiatry.

[7] Kreitman N, Casey P (1988). Repetition of parasuicide: an epidemiological and clinical study. Br J Psychiatry.

[8] Mendez-Bustos P, De Leon-Martinez V, Miret M, Baca-Garcia E, Lopez-Castroman J (2013). Suicide reattempters: A systematic review. Harv Rev Psychiatry.

[9] Parra-Uribe I, Blasco-Fontecilla H, Garcia-Parés G, Martínez-Naval L, Valero-Coppin O, Cebrià-Meca A (2017). Risk of re-attempts and suicide death after a suicide attempt: a survival analysis. BMC Psychiatry.

[10] Lengvenyte A, Olié E, Strumila R, Navickas A, Gonzalez Pinto A, Courtet P. Immediate and short-term efficacy of suicide-targeted interventions in suicidal individuals: a systematic review. World J Biol Psychiatry. 2021;22:670–85. 10.1080/15622975.2021.1907712 33783294

[11] Lopez-Castroman J, Jaussent I, Gorwood P, Courtet P (2016). Suicidal depressed patients respond less well to antidepressants in the short term. Depress Anxiety.

[12] Courtet P, Lopez-Castroman J (2017). Antidepressants and suicide risk in depression. World Psychiatry.

[13] Sutherland JJ, Daly TM, Liu X, Goldstein K, Johnston JA, Ryan TP (2015). Co-prescription trends in a large cohort of subjects predict substantial drug-drug interactions. PLoS ONE.

[14] Payne RA (2016). The epidemiology of polypharmacy. Clin Med.

[15] Shehab N, Lovegrove MC, Geller AI, Rose KO, Weidle NJ, Budnitz DS (2016). US emergency department visits for outpatient adverse drug events, 2013–2014. JAMA.

[16] Kessler C, Ward MJ, McNaughton CD (2016). Reducing adverse drug events: the need to rethink outpatient prescribing. JAMA.

[17] Goffin KC, Dell’Osso B, Miller S, Wang PW, Holtzman JN, Hooshmand F (2016). Different characteristics associated with suicide attempts among bipolar I versus bipolar II disorder patients. J Psychiatr Res.

[18] Dell’osso B, Vismara M, Dobrea C, Cremaschi L, Grancini B, Arici C (2018). Clinical characterization of Italian suicide attempters with bipolar disorder. CNS Spectr.

[19] Dold M, Bartova L, Mendlewicz J, Souery D, Serretti A, Porcelli S (2018). Clinical correlates of augmentation/combination treatment strategies in major depressive disorder. Acta Psychiatr Scand.

[20] Sim SC, Kacevska M, Ingelman-Sundberg M (2013). Pharmacogenomics of drug-metabolizing enzymes: a recent update on clinical implications and endogenous effects. Pharmacogenom J.

[21] Meltzer HY, Baldessarini RJ (2003). Reducing the risk for suicide in schizophrenia and affective disorders. J Clin Psychiatry.

[22] Rich CL, Isaccson G (2003). Treatment of anxiety in suicidal patients. J Clin Psychiatry.

[23] Rau T, Wohlleben G, Wuttke H, Thuerauf N, Lunkenheimer J, Lanczik M (2004). CYP2D6 genotype: impact on adverse effects and nonresponse during treatment with antidepressants-a pilot study. Clin Pharm Ther.

[24] Kawanishi C, Lundgren S, Agren H, Bertilsson L (2004). Increased incidence of CYP2D6 gene duplication in patients with persistent mood disorders: ultrarapid metabolism of antidepressants as a cause of nonresponse. A pilot study. Eur J Clin Pharmacol.

[25] Lobello KW, Preskorn SH, Guico-Pabia CJ, Jiang Q, Paul J, Nichols AI (2010). Cytochrome P450 2D6 phenotype predicts antidepressant efficacy of venlafaxine: a secondary analysis of 4 studies in major depressive disorder. J Clin Psychiatry.

[26] Tsai M-H, Lin K-M, Hsiao M-C, Shen WW, Lu M-L, Tang H-S (2010). Genetic polymorphisms of cytochrome P450 enzymes influence metabolism of the antidepressant escitalopram and treatment response. Pharmacogenomics.

[27] Peñas-Lledó EM, Trejo HD, Dorado P, Ortega A, Jung H, Alonso E (2013). CYP2D6 ultrarapid metabolism and early dropout from fluoxetine or amitriptyline monotherapy treatment in major depressive patients. Mol Psychiatry.

[28] Jukić MM, Haslemo T, Molden E, Ingelman-Sundberg M (2018). Impact of CYP2C19 genotype on escitalopram exposure and therapeutic failure: a retrospective study based on 2,087 patients. Am J Psychiatry.

[29] Zackrisson AL, Lindblom B, Ahlner J (2010). High frequency of occurrence of CYP2D6 gene duplication/multiduplication indicating ultrarapid metabolism among suicide cases. Clin Pharmacol Ther.

[30] Peñas-Lledó EM, Dorado P, Agüera Z, Gratacós M, Estivill X, Fernández-Aranda F (2011). High risk of lifetime history of suicide attempts among CYP2D6 ultrarapid metabolizers with eating disorders. Mol Psychiatry.

[31] Stingl JC, Viviani R (2011). CYP2D6 in the brain: impact on suicidality. Clin Pharmacol Ther.

[32] Peñas-Lledó EM, Blasco-Fontecilla H, Dorado P, Vaquero-Lorenzo C, Baca-García E, Llerena A (2012). CYP2D6 and the severity of suicide attempts. Pharmacogenomics.

[33] Persson A, Sim SC, Virding S, Onishchenko N, Schulte G, Ingelman-Sundberg M (2014). Decreased hippocampal volume and increased anxiety in a transgenic mouse model expressing the human CYP2C19 gene. Mol Psychiatry.

[34] Jukić MM, Opel N, Ström J, Carrillo-Roa T, Miksys S, Novalen M (2017). Elevated CYP2C19 expression is associated with depressive symptoms and hippocampal homeostasis impairment. Mol Psychiatry.

[35] Sim SC, Nordin L, Andersson TM-L, Virding S, Olsson M, Pedersen NL (2010). Association between CYP2C19 polymorphism and depressive symptoms. Am J Med Genet B Neuropsychiatr Genet.

[36] Crosby AE, Ortega LV, Melanson C. Self-directed violence surveillance: uniform definitions and recommended data elements, Version 1.0. Atlanta (GA): Centers for Disease Control and Prevention, National Center for Injury Prevention and Control; 2011. https://www.cdc.gov/suicide/pdf/self-directed-violence-a.pdf.

[37] Asberg M, Träskman L, Thorén P (1976). 5-HIAA in the cerebrospinal fluid. A biochemical suicide predictor?. Arch Gen Psychiatry.

[38] Coccaro EF, Berman ME, Kavoussi RJ (1997). Assessment of life history of aggression: development and psychometric characteristics. Psychiatry Res.

[39] Beck AT, Kovacs M, Weissman A (1979). Assessment of suicidal intention: the scale for suicide ideation. J Consulting Clin Psychol.

[40] Stefansson J, Nordström P, Jokinen J (2012). Suicide intent scale in the prediction of suicide. J Affect Disord.

[41] Beck AT, Schuyler D, Herman I. Development of suicidal intent scales. In: Beck AT, Resnik HLP, Lettieri DJ, editors. The Prediction of Suicide. Philadelphia: Charles Press; 1974. pp. 45–56.

[42] Dorado P, Cáceres MC, Pozo-Guisado E, Wong M-L, Licinio J, Llerena A (2005). Development of a PCR-based strategy for CYP2D6 genotyping including gene multiplication of worldwide potential use. Biotechniques.

[43] Gaedigk A, Simon SD, Pearce RE, Bradford LD, Kennedy MJ, Leeder JS (2008). The CYP2D6 activity score: translating genotype information into a qualitative measure of phenotype. Clin Pharmacol Ther.

[44] Llerena A, Dorado P, Ramírez R, González I, Alvarez M, Peñas-Lledó EM (2012). CYP2D6 genotype and debrisoquine hydroxylation phenotype in Cubans and Nicaraguans. Pharmacogenom J.

[45] Mrazek DA, Biernacka JM, O’Kane DJ, Black JL, Cunningham JM, Drews MS (2011). CYP2C19 variation and citalopram response. Pharmacogenet Genom.

[46] Villagra D, Goethe J, Schwartz HI, Szarek B, Kocherla M, Gorowski K (2011). Novel drug metabolism indices for pharmacogenetic functional status based on combinatory genotyping of CYP2C9, CYP2C19 and CYP2D6 genes. Biomark Med.

[47] Peñas-Lledó E, Guillaume S, Naranjo MEG, Delgado A, Jaussent I, Blasco-Fontecilla H (2015). A combined high CYP2D6-CYP2C19 metabolic capacity is associated with the severity of suicide attempt as measured by objective circumstances. Pharmacogenom J.

[48] Wei S, Liu L, Bi B, Li H, Hou J, Tan S (2013). An intervention and follow-up study following a suicide attempt in the emergency departments of four general hospitals in Shenyang, China. Crisis.

[49] Hultén A, Jiang G-X, Wasserman D, Hawton K, Hjelmeland H, de Leo D (2001). Repetition of attempted suicide among teenagers in Europe: frequency, timing and risk factors. Eur Child Adolesc Psychiatry.

[50] Hawton K, Houston K, Haw C, Townsend E, Harriss L (2003). Comorbidity of Axis I and Axis II disorders in patients who attempted suicide. Am J Psychiatry.

[51] Pagura J, Cox BJ, Sareen J, Enns MW (2008). Factors associated with multiple versus single episode suicide attempts in the 1990–1992 and 2001–2003 United States National Comorbidity Surveys. J Nerv Ment Dis.

[52] Miller TR, Swedler DI, Lawrence BA, Ali B, Rockett IRH, Carlson NN (2020). Incidence and lethality of suicidal overdoses by drug class. JAMA Netw Open.

